# A Modified Integrated Genetic Model for Risk Prediction in Younger Patients with Acute Myeloid Leukemia

**DOI:** 10.1371/journal.pone.0153016

**Published:** 2016-04-06

**Authors:** Caroline E. Sloan, Marlise R. Luskin, Anne M. Boccuti, Alison R. Sehgal, Jianhua Zhao, Robert D. Daber, Jennifer J. D. Morrissette, Selina M. Luger, Adam Bagg, Phyllis A. Gimotty, Martin Carroll

**Affiliations:** 1 Department of Medicine, Perelman School of Medicine at the University of Pennsylvania, Philadelphia, Pennsylvania, United States of America; 2 Department of Pathology and Laboratory Medicine, Perelman School of Medicine at the University of Pennsylvania, Philadelphia, Pennsylvania, United States of America; 3 Division of Hematology and Oncology, Perelman School of Medicine at the University of Pennsylvania, Philadelphia, Pennsylvania, United States of America; 4 Department of Biostatistics and Epidemiology, Perelman School of Medicine at the University of Pennsylvania, Philadelphia, Pennsylvania, United States of America; 5 Division of Hematology and Oncology, University of Pittsburgh School of Medicine, Pittsburgh, Pennsylvania, United States of America; 6 Philadelphia Veterans Administration Medical Center, Philadelphia, Pennsylvania, United States of America; Queen's University Belfast, UNITED KINGDOM

## Abstract

**Background:**

Although cytogenetics-based prognostication systems are well described in acute myeloid leukemia (AML), overall survival (OS) remains highly variable within risk groups. An integrated genetic prognostic (IGP) model using cytogenetics plus mutations in nine genes was recently proposed for patients ≤60 years to improve classification. This model has not been validated in clinical practice.

**Methods and Findings:**

We retrospectively studied 197 patients with newly diagnosed de novo AML. We compared OS curves among the mutational profiles defined by the IGP model. The IGP model assigned patients with intermediate cytogenetics as having favorable, intermediate or unfavorable mutational profiles. The IGP model reassigned 50 of 137 patients with intermediate cytogenetics to favorable or unfavorable mutational profiles. Median OS was 2.8 years among 14 patients with intermediate cytogenetics and favorable mutational profiles (mutant *NPM1* and mutant *IDH1* or *IDH2*) and 1.3 years among patients with intermediate mutational profiles. Among patients with intermediate cytogenetics labeled as having unfavorable mutational profiles, median OS was 0.8 years among 24 patients with *FLT3*-ITD positive AML and high-risk genetic changes (trisomy 8, *TET2* and/or *DNMT3A*) and 1.7 years among 12 patients with *FLT3*-ITD negative AML and high-risk mutations (*TET2*, *ASXL1* and/or *PHF6*). OS for patients with intermediate cytogenetics and favorable mutational profiles was similar to OS for patients with favorable cytogenetics (p = 0.697) and different from patients with intermediate cytogenetics and intermediate mutational profiles (p = 0.028). OS among patients with *FLT3*-ITD positive AML and high-risk genetic changes was similar to patients with unfavorable cytogenetics (p = 0.793) and different from patients with intermediate IGP profile (p = 0.022). Patients with *FLT3*-ITD negative AML and high-risk mutations, defined as ‘unfavorable’ in the IGP model, had OS similar to patients with intermediate IGP profile (p = 0.919).

**Conclusions:**

The IGP model was not completely validated in our cohort. However, mutations in six out of the nine genes can be used to characterize survival (*NPMI*, *IDH1*, *IDH2*, *FLT3-*ITD, *TET2*, *DNMT3A*) and allow for more robust prognostication in the patients who are re-categorized by the IGP model. These mutations should be incorporated into clinical testing for younger patients outside of clinical trials, in order to guide therapy.

## Introduction

Acute myeloid leukemia (AML) is a heterogeneous disease with a wide range of clinical outcomes. Clinicians have traditionally relied on clinical features of the patient and disease, as well as the pre-treatment karyotype of leukemic blasts, to predict a patient’s clinical outcome [1,2]. While the presence of a favorable- or unfavorable-risk karyotype may provide useful prognostic information, the outcomes of patients with intermediate cytogenetics—the largest subgroup—remain highly heterogeneous, making treatment planning challenging. Recently, the prognostic relevance of mutations in *FLT3*, *NPM1* and *CEBPA* have been established in patients aged ≤60 years old, specifically in those with intermediate cytogenetic risk [3–6]. These mutations have been incorporated into AML prognostic schemas and are recommended for standard testing in patients with newly diagnosed AML [7–9]. Despite these advances, our ability to predict clinical outcomes in patients with AML, especially those with intermediate-risk cytogenetics, remains limited [2,3,8].

In recent years, advances in sequencing technology have led to the rapid identification of additional recurrent somatic mutations in AML. Mutations in several genes, including *TET2*, *DNMT3A*, *ASXL1* and *PHF6*, have been associated with poor prognosis in some cohorts [10–15], while the prognostic impact of mutations in other genes, such as *IDH1* and *IDH2*, is less clear [16]. In 2012, Patel *et al*. proposed a prognostic model that integrates cytogenetics and mutational status for nine genes [14]. This model was developed using data from patients enrolled in a multicenter Eastern Cooperative Oncology Group (ECOG) trial of different doses of anthracycline. Despite the use of this prognostic model by some, its validity in clinical practice has not been demonstrated. The aim of this study was to evaluate the performance of Patel *et al*.’s integrated prognostic model in a cohort of patients with de novo AML seen in clinical practice, and to investigate refinements that could improve risk prediction.

## Methods

### Patient cohort

This retrospective study included 197 patients with de novo AML aged ≤60 years who were treated with intensive induction chemotherapy at the University of Pennsylvania (Penn) or Washington University between 2001 and 2013. De novo AML was defined as no prior diagnosis of hematologic malignancy or myelodysplastic syndrome, or history of previous chemotherapy or radiation. The 106 patients from Penn provided written informed consent to donation of a diagnostic bone marrow, peripheral blood, or pheresis sample to the institution’s Hematologic Malignancies Tissue Bank. Information about patient and disease characteristics, treatment, and clinical outcome was obtained from review of the medical records. We note that 32 of the Penn patients were included in the ECOG 1900 study, which was used by Patel *et al*. to develop their integrated model. The 91 patients from Washington University were identified from The Cancer Genome Atlas (TCGA), which includes data on clinical and disease characteristics, as well as patient outcomes [17]. Patients with karyotype t(15;17), unknown cytogenetic risk, or unavailable clinical information were excluded. All patients received induction chemotherapy at their respective institutions with cytarabine and an anthracycline [18]. Patients did not all get the same dose of induction chemotherapy, reflecting management differences seen in clinical practice at the time patients were treated. Post-induction chemotherapy and allogeneic stem cell transplant (SCT) regimens were varied and provided at physicians’ discretion. Overall survival (OS) was the primary endpoint and was defined as the time between AML diagnosis and death. Patients alive at the time of data collection were censored at the date of last follow-up. The Penn institutional review board approved this study.

### Genetic analysis

Cytogenetic risk was classified as favorable, intermediate, or unfavorable according to the modified Medical Research Council (MRC) criteria [2]. Mutational analysis of samples from Penn was performed by the Center for Personalized Diagnostics, using an amplicon-based custom targeted next-generation sequencing panel for 33 hematologic malignancy-associated genes (TruSeq Custom Amplicon, Illumina Inc.; S1 Table). The libraries generated were pooled and sequenced on the Illumina MiSeq (Illumina, Inc.). The mean depth of coverage across the entire panel was 2000x to achieve a minimum read depth of 250x at any given position. Variant allele frequency was consistently detectable down to 4%. All of the genes included in the panel used by Patel *et al*., except for *MLL*-PTD and *CEBPA*, were included in the Penn multiplex panel. The *CEBPA* gene was isolated using long range PCR, prepared for sequencing using the Nextera library preparation kit (Illumina, Inc.), and sequenced in tandem with the hematologic next-generation sequencing panel (manuscript in preparation). At Washington University, whole-genome or whole-exome sequencing was performed on all samples with matched normal skin samples, as previously described [17]. Validation re-sequencing for all of the TCGA samples was performed at Wash U. The minimum variant allele frequency for single nucleotide variants was 8%, which was the standard value when using VarScan2 at the time (except for CEBPA, which was 5%, because coverage was lower for that locus).

### Statistical analysis

Differences in dichotomous variables between groups were compared using Pearson’s chi-square statistic or Fisher’s exact test; differences in continuous variables between groups were compared using the median score test. Kaplan-Meier OS curves were computed and compared using the log-rank statistic. Adjusted p-values were computed using Sidak’s procedure for pairwise comparisons after a significant log-rank test when there were more than two groups. A classification model was considered to be valid if there was a significant difference between the OS curves of each risk group defined by the model. Unadjusted and adjusted hazard ratios (HR) were estimated using univariate and multivariate Cox proportional hazards regression models. The reduced multivariate model was developed by backward sequential elimination of the least non-significant factor in the model to develop the most parsimonious model. SAS version 9.4 was used for statistical analysis. R was used to produce graphs.

## Results

### Patient characteristics

Patient and disease characteristics, including mutational profile, were similar for the Penn and TCGA cohorts, with the exception of hemoglobin, peripheral blast percentage and bone marrow blast percentage (Tables 1 and 2). Among patients at Penn, median follow-up time was 5.1 years for those alive at the end of the study and 1.3 years for those who died. Among patients at Washington University, median follow-up was 2.1 years for those alive at the end of the study and 1.0 year for those who died. The two cohorts were combined for further analysis. The median age of the overall study cohort was 49 years (range, 18–60 years) and 55% were male. The majority had intermediate cytogenetics (70%) and the three most frequent mutations were *NPM1* (34%), *DNMT3A* (30%) and *FLT3*-ITD (26%). Median follow-up time for the study cohort was 3.0 years among patients alive at the end of the study and 1.0 year among patients who died.

**Table 1:** Clinical characteristics.

	Penn (n = 106)	TCGA (n = 91)	Penn vs. TCGA	Penn + TCGA (n = 197)
*Clinical characteristics*	*%*	*%*	*P*	*% (95% CI)*
Male	57	53	0.67	55 (48–62)
Race			0.81	
White	76	90		82 (76–87)
Nonwhite	9	9		9 (5–14)
Unknown	16	1		9 (6–14)
Cytogenetic profile (MRC)			0.91	
Favorable	15	13		14 (9–19)
Intermediate	70	69		70 (63–76)
Unfavorable	15	18		16 (11–21)
Transplant	55	68	0.06	61 (54–68)
*Clinical characteristics*	*Median (range)*	*Median (range)*	*P*	*Median (range)*
Age at diagnosis	50 (18–60)	47 (18–60)	0.27	49 (18–60)
WBC, x10^9^/L	33 (1–305)	22 (1–298)	0.13	29 (1–305)
Hemoglobin, g/dL	8.8 (3–13)	9.0 (6–13)	0.01	9.0 (3–13)
Platelets, g/dl	50 (6–613)	56 (9–351)	0.70	52 (6–613)
Peripheral blasts, %	50 (0–96)	72 (0–100)	<0.01	62 (0–100)
Bone marrow blasts, %	82 (19–100)	72 (0–97)	0.02	77 (0–100)

Penn: Hospital of the University of Pennsylvania; TCGA: The Cancer Genome Network; 95% CI: 95% confidence interval; MRC: Modified Medical Research Council; WBC: white blood cell count.

**Table 2:** Genetic characteristics.

Mutational frequency	Penn (n = 106)	TCGA (n = 91)	Penn vs. TCGA	Penn + TCGA (n = 197)
	*%*	*%*	*P*	*% (95% CI)*
*NPM1*	34	34	1.00	34 (27–41)
*DNMT3A*	33	27	0.44	30 (24–37)
*FLT3*			0.15	
*FLT3*-ITD	34	18	—	26 (20–33)
*FLT3*-TKD	5	10	—	7 (4–11)
*FLT3* other^a^	1	1	—	1 (0–4)
*FLT3* total^b^	39	29	—	34 (27–41)
*TET2*	9	8	0.80	9 (5–13)
*IDH1*	9	13	0.50	11 (7–16)
*CEBPA*	8	11	0.46	9 (5–13)
*RUNX1*	8	5	0.77	7 (3–10)
*IDH2*	9	4	0.27	7 (4–11)
*ASXL1*	4	1	0.38	3 (1–5)
*WT1*	5	9	0.27	7 (3–10)
*TP53*	5	4	1.00	5 (2–7)
*KIT*	4	5	1.00	5 (2–8)
*PHF6*	0	4	0.42	3 (1–5)

Penn: Hospital of the University of Pennsylvania; TCGA: The Cancer Genome Network.

^a^ ‘*FLT3* other’ includes all *FLT3* mutations in our database that were not categorized as *FLT3*-ITD or *FLT3*-TKD (N841K, D839G, M578T).

^b^ ‘*FLT3* total’ includes all *FLT3* mutations

Overall, the characteristics of the study cohort, including mutational profile, were similar to the characteristics of the ECOG 1900 cohort that Patel *et al*. used to develop their model [14]. Median survival time in the ECOG 1900 cohort, defined by Patel *et al*. as time to death for those who died or time to last follow-up for those alive at the time of analysis, was 1.7 years at the time of reporting [14].

### Overall survival by cytogenetic risk

As expected, OS curves varied by cytogenetic risk. OS for patients with favorable cytogenetics was significantly better than OS for patients with unfavorable cytogenetics (adjusted p = 0.003; S1 Fig). Of note, there was no significant difference in OS curves between patients with favorable and intermediate cytogenetics (adjusted p = 0.141), or between patients with intermediate and unfavorable cytogenetics (adjusted p = 0.976).

### Overall survival by integrated genetic prognostic profile

**Fig 1:**
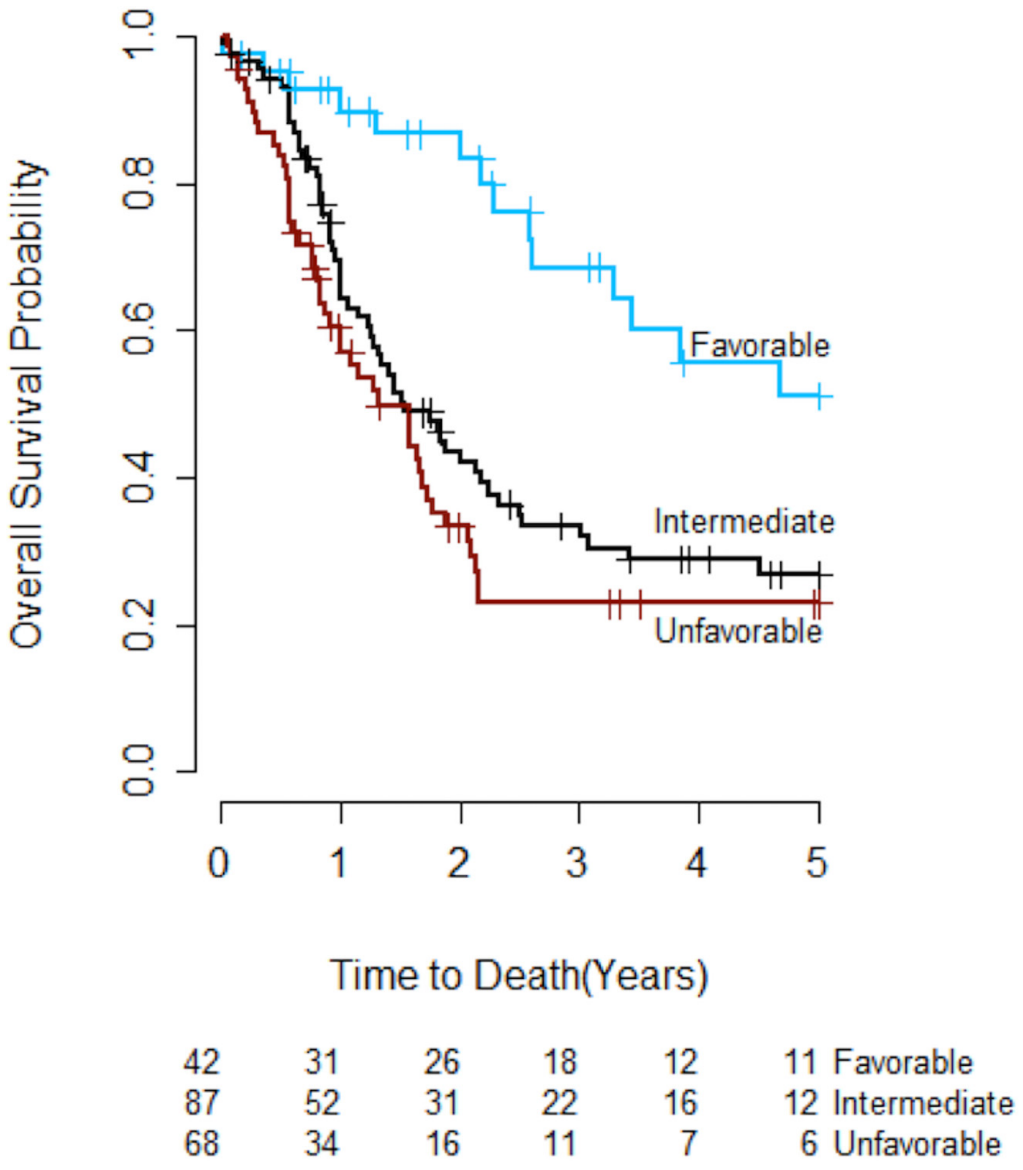
Overall survival by integrated genetic prognostic (IGP) profile (n = 197). The overall survival curve for patients with favorable IGP risk was significantly different from the curve for patients with unfavorable IGP risk (adjusted p<0.001). There was no significant difference in survival curves between patients with favorable IGP risk and patients with intermediate IGP risk (adjusted p = 0.055), or between patients with unfavorable IGP risk and patients with intermediate IGP risk (adjusted p = 0.596).

The integrated genetic prognostic model developed by Patel *et al*., (hereafter referred to as the IGP model; Table 3) was evaluated to determine whether the risk groups it defined had different OS in our study cohort. The IGP model reassigned 14 patients with intermediate cytogenetics to the favorable risk group and 36 to the unfavorable risk group, with the remaining 87 patients classified as having intermediate IGP risk. After reclassification, 21% (42), 44% (87), and 35% (68) of the study patients were categorized as having favorable, intermediate and unfavorable IGP risk, respectively (Table 3). The OS curves for the three IGP risk groups are presented in Fig 1. OS in the favorable IGP risk group was significantly better than OS in the unfavorable IGP risk group (adjusted p<0.001), but there was no significant difference in OS between patients with favorable and intermediate IGP risk (adjusted p = 0.055), or between patients with unfavorable and intermediate IGP risk (adjusted p = 0.596).

**Table 3:** Schematic representation of integrated genetic prognostic (IGP) model and modified IGP model.

Cytogenetic classification (n, %)	Mutational profile			Integrated genetic prognostic (IGP) model		Modified IGP model (n, %)
	*CEBPA*	*FLT3*-ITD	Other mutations	Subgroup (n, %)	Profile (n, %)	
Favorable (28, 14%)	Any			28 (14%)	Favorable (42, 21%)	Favorable (42, 21%)
	Negative	Negative	*NPM1*+ and *IDH1/IDH2*+	14 (7%)		
	Negative	Negative	*MLL*-PTD-, *TET2*-, *ASXL1-*and *PHF6-*			
	Positive	Any	Any	87 (44%)	Intermediate (87, 44%)	
Intermediate (137, 70%)	Negative	Positive	Trisomy 8-, *MLL*-PTD-, *TET2*- and *DNMT3A*-			Intermediate (99, 50%)
	Negative	Negative	*MLL*-PTD+, *TET2+*, *ASXL1+* and/or *PHF6+*	12 (6%)		
	Negative	Positive	Trisomy 8, *MLL*-PTD+, *TET2*+ and/or *DNMT3A*+	24 (12%)	Unfavorable (68, 35%)	Unfavorable (56, 28%)
Unfavorable (32, 16%)	Any			32 (16%)		

Adapted from Patel *et al*.[14] “+” = abnormality or mutation present; “-” = abnormality or mutation absent.

**Fig 2:**
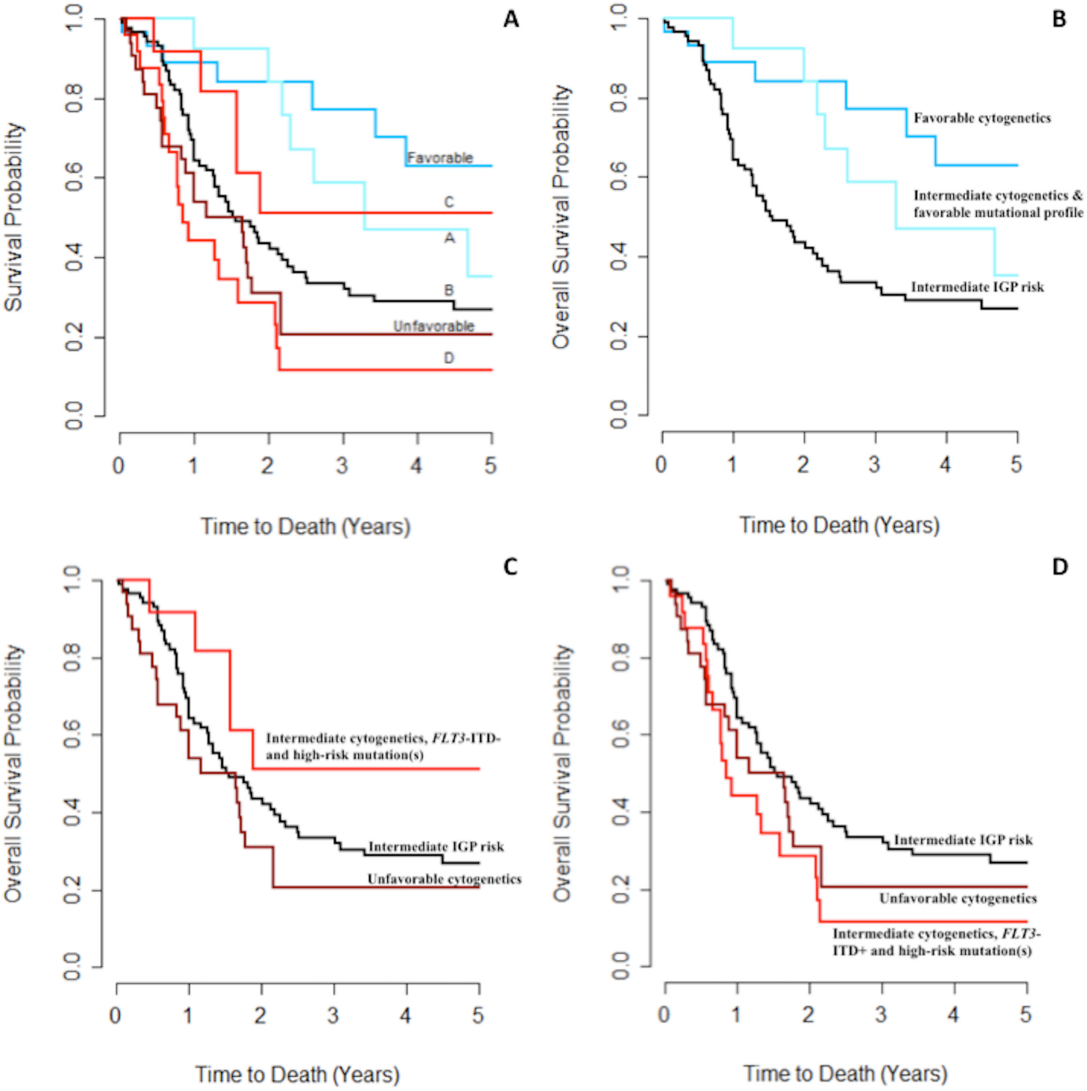
Overall survival in IGP model subgroups. A. Overall survival by cytogenetics and mutational profiles. Among patients with intermediate cytogenetics, three-year overall survival was 59% for those with favorable mutational profiles (A), 33% for those with intermediate mutational profiles (B), 51% for those who were *FLT3-*ITD negative with high-risk mutations (C) and 11% for those who were *FLT3-*ITD positive with high-risk mutations (D). Three-year overall survival was 77% among patients with favorable cytogenetics (favorable) and 21% among patients with unfavorable cytogenetics (unfavorable). B. Overall survival among patients with favorable mutational profiles. The overall survival curve for patients with intermediate cytogenetics and mutant *NPM1* plus mutant *IDH1* or *IDH2* was similar to the survival curve for patients with favorable cytogenetics (adjusted p = 0.697) and different from the survival curve for patients in the intermediate IGP risk group (adjusted p = 0.028). C. Overall survival among patients with *FLT3-*ITD negative AML and high-risk mutations. The overall survival curve for patients with *FLT3*-ITD negative (*FLT3-*ITD-) AML and co-occurring high-risk mutations (*TET2*, *ASXL1* and/or *PHF6*) was not significantly different from the survival curves for patients with unfavorable cytogenetics or patients with intermediate IGP risk (adjusted p = 0.111 and p = 0.919, respectively). D. Overall survival among patients with *FLT3*-ITD positive AML and high-risk mutations. The overall survival curve for patients with *FLT3*-ITD positive (*FLT3*-ITD+) AML and co-occurring high-risk mutations (trisomy 8, *TET2* and/or *DNMT3A*) was similar to the survival curve for patients with unfavorable cytogenetics (adjusted p = 0.793) and different from the survival curve for patients in the intermediate IGP risk group (adjusted p = 0.022).

Survival among subgroups of patients who were reclassified in the IGP model was further examined in order to better understand the impact of molecularly defined genetic mutations on prognosis (Fig 2). Fig 2A presents the OS curves for the six cytogenetic and mutational profiles that comprise the IGP model and is shown as a summary for comparison of all groups. Reclassification of a subgroup of patients was considered to be successful when the OS curve of the reclassified subgroup was 1) similar to the OS curve of the risk group to which it was reclassified, and 2) different from the OS curve of the group from which it was removed, i.e. the intermediate IGP risk group. These subgroups were analyzed in more detail, as discussed below.

#### Favorable mutational profile: NPM1 mutant plus IDH1/IDH2 mutant

The OS curve for patients in the study cohort who had intermediate cytogenetics and mutant *NPM1* plus mutant *IDH1* or *IDH2* (*NPM1*mut/*IDH*mut) was similar to the OS curve for patients with favorable cytogenetics (adjusted p = 0.697) and was significantly different from the OS curve for patients in the intermediate IGP risk group (adjusted p = 0.028; Fig 2B). Closer examination shows that while the OS curve for patients with *NPM1*mut/*IDH*mut was similar to the OS curve for patients with favorable cytogenetics in the first two years, it was closer to the OS curve for the intermediate IGP risk group after two years. Nine of the 14 patients in this group relapsed, up to three years after the initial diagnosis.

#### Unfavorable mutational profiles

Two subgroups of patients with intermediate cytogenetics were reclassified to the unfavorable IGP risk group. The OS curve for the 12 patients in the first subgroup–*FLT3*-ITD negative AML with co-occurring high-risk mutations as defined by the IGP model (*TET2*, *ASXL1* and/or *PHF6*)–was not significantly different from the OS curves for patients with unfavorable cytogenetics or patients with intermediate IGP risk (adjusted p = 0.111 and p = 0.919, respectively; Fig 2C). In contrast, the OS curve for the 24 patients in the second subgroup–*FLT3*-ITD positive AML with co-occurring high-risk genetic changes (trisomy 8, *TET2* and/or *DNMT3A*)–was similar to the OS curve for patients with unfavorable cytogenetics (adjusted p = 0.793) and significantly different from the OS curve for the intermediate IGP risk group (adjusted p = 0.022; Fig 2D).

### Modified IGP model

Based on these observations, a modified IGP (M-IGP) model was developed (Table 3, far right column). The 14 patients with intermediate cytogenetics and favorable mutational profiles were reclassified as having favorable prognosis. The 24 patients with intermediate cytogenetics who were *FLT3*-ITD positive and had co-occurring high-risk mutations (12% of total; 18% of those with intermediate cytogenetics) were reclassified as having unfavorable prognosis. Overall, 21% (42), 50% (99) and 28% (56) of the study patients were categorized as having favorable, intermediate and unfavorable M-IGP risk, respectively (Table 3). Using this model, the OS curves were significantly different between patients with favorable and unfavorable M-IGP profiles (adjusted p<0.001), but there was again no significant difference in OS between patients with favorable and intermediate M-IGP profiles (adjusted p = 0.178) or between patients with unfavorable and intermediate M-IGP profiles (adjusted p = 0.100).

**Fig 3:**
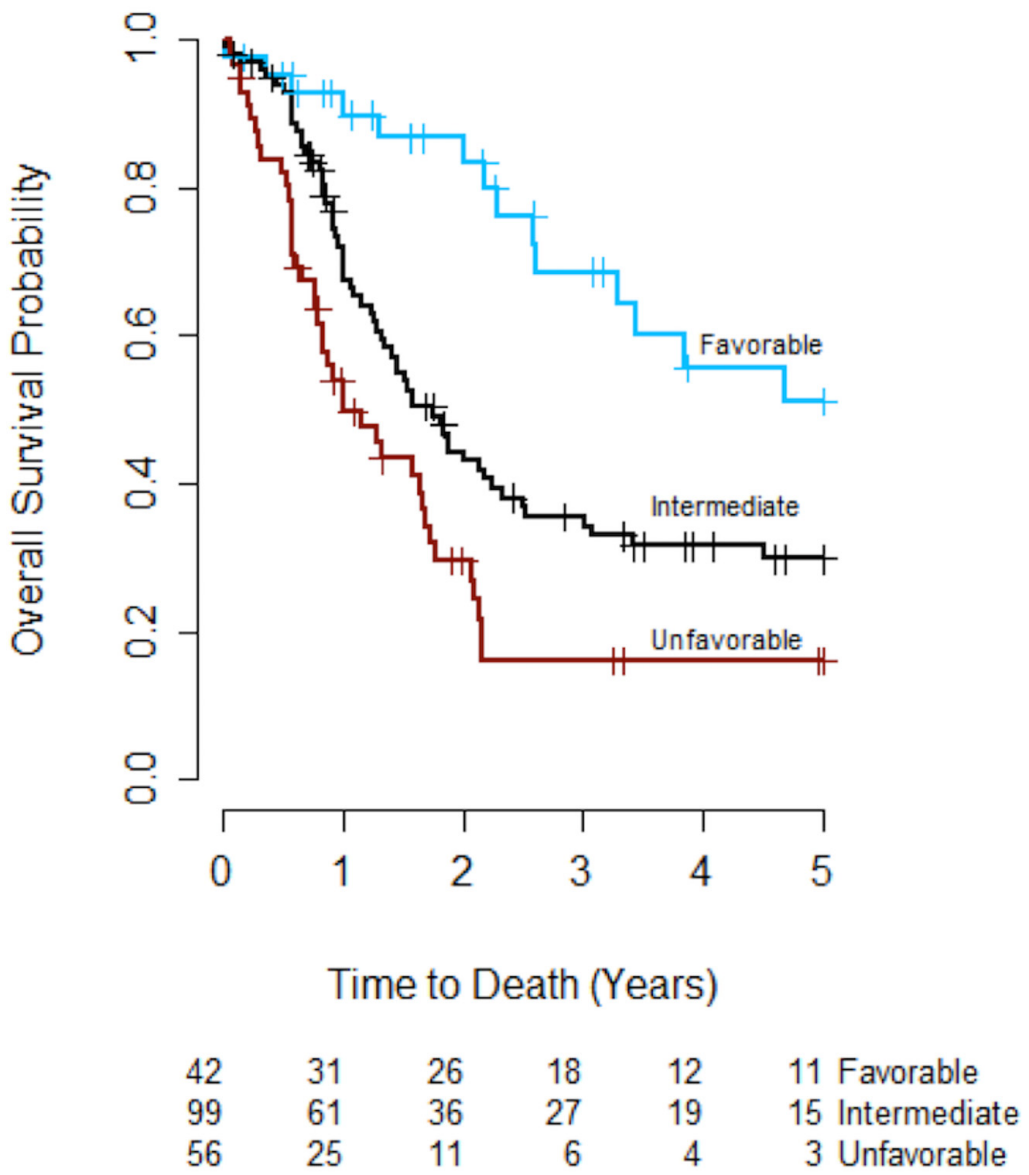
Overall survival by modified IGP profile (n = 197). The overall survival curve for patients with favorable M-IGP risk was significantly different from the survival curve for patients with unfavorable IGP risk (adjusted p<0.001). There was no significant difference in survival curves between patients with favorable M-IGP profiles and patients with intermediate M-IGP profiles (adjusted p = 0.178), or between patients with unfavorable M-IGP profiles and patients with intermediate M-IGP profiles (adjusted p = 0.100).

Three-year OS rates were 69%, 36% and 16% among patients with favorable, intermediate and unfavorable M-IGP profiles, respectively (Table 4 and Fig 3). The absolute difference in three-year OS rates between favorable and unfavorable profiles in the study cohort was larger in the M-IGP model (53%) than in the IGP (46%) model, but this difference was largest in the cytogenetic model (56%).

**Table 4:** Three-year overall survival by classification system.

	3-year overall survival, % (95% CI)		
Prognostic group	Cytogenetic classification	Integrated genetic prognostic (IGP) model	Modified IGP model
**Favorable**	77 (58–96)	69 (52–85)	69 (52–85)
**Intermediate**	34 (25–42)	33 (23–44)	36 (25–46)
**Unfavorable**	21 (4–37)	23 (12–34)	16 (5–27)

95% CI: 95% confidence interval

#### Univariate and multivariate analyses

The correlations between clinical and genetic variables and prognosis were considered next. The only factor that was significant in the univariate Cox regression analyses was the M-IGP classification (Table 5). Compared to patients in the intermediate M-IGP risk group, patients with favorable M-IGP risk had significantly better prognosis (HR = 0.47, 95% CI = 0.23–0.94, p = 0.034) and patients with unfavorable M-IGP risk had significantly worse prognosis (HR = 2.07, 95% CI = 1.40–3.05, p<0.001). M-IGP profile remained a significant prognostic factor in the multivariate model that controlled for clinical factors and treatment site, and was the only significant factor in the reduced multivariate model.

**Table 5:** Univariate and multivariate Cox models for overall survival (n = 197).

	Univariate model		Multivariate model		Reduced model	
	Hazard ratio (95% CI)	P	Hazard ratio (95% CI)	P	Hazard ratio (95% CI)	P
**Age group**						
18–29	0.73 (0.40–1.32)	0.291	0.75 (0.36–1.58)	0.454	---	---
30–39	0.79 (0.48–1.31)	0.357	0.77 (0.44–1.35)	0.360		
40–49	1.05 (0.65–1.70)	0.855	1.34 (0.78–2.30)	0.291		
50–60	---	---	---	---		
**Sex**						
Female	1.12 (0.78–1.60)	0.547	0.97 (0.65–1.46)	0.882	---	---
Male	---	---	---	---		
**M-IGP model**						
Favorable	0.47 (0.23–0.94)	0.034	0.68 (0.32–1.45)	0.318	0.47 (0.23–0.94)	0.034
Intermediate	---	---	---	---	---	---
Unfavorable	2.07 (1.40–3.05)	<0.001	2.14 (1.38–3.48)	0.001	2.07 (1.40–3.05)	<0.001
**Site**						
Penn	1.08 (0.75–1.55)	0.697	0.93 (0.57–1.52)	0.770	---	---
TCGA	---	---	---	---		
**White blood cell count**	1.003 (1.00–1.01)	0.077	1.00 (0.998–1.01)	0.224		---
**Hemoglobin**	1.04 (0.93–1.16)	0.532	1.02 (0.88–1.17)	0.828		---
**Platelets**	1.001 (0.998–1.00)	0.485	1.00 (0.998–1.01)	0.300		---
**Peripheral blasts**	1.004 (0.998–1.01)	0.165	1.00 (0.99–1.01)	0.918		---
**Bone marrow blasts**	1.006 (0.998–1.01)	0.152	1.01 (0.99–1.02)	0.268		---

95% CI: 95% confidence interval; M-IGP model: Modified integrated genetic prognostic model

## Discussion

Every AML diagnosis is accompanied by an assessment of prognosis, customarily based on the clinical features of the patient as well as pre-treatment karyotype [1,19]. Prognostication not only helps set accurate patient expectations, but also informs recommendations for post-remission therapy, including allogeneic SCT. Most experts recommend that patients with favorable cytogenetics proceed with chemotherapy-based consolidation [9,18]. In contrast, patients with unfavorable cytogenetics or other high-risk clinical features should undergo SCT in first remission, because of their poor chance of achieving cure with chemotherapy alone. An optimal treatment course for patients with intermediate cytogenetics has not been defined, however [18]. These patients have highly varied clinical outcomes, making it difficult to provide an assessment of prognosis or informed recommendations for consolidation therapy.

Recently, a number of recurrent somatic mutations associated with AML have been shown to be independently associated with prognosis [5,11,20–25]. The challenge is now to incorporate the prognostic information of these frequently co-occurring mutations with established clinical and cytogenetic factors, in order to improve our ability to determine AML prognosis and improve clinical care, particularly among patients with intermediate cytogenetics. The European LeukemiaNet (ELN) was the first group to develop a prognostic model that accounted for both cytogenetics and molecular markers. This model was validated in a large European cohort [8], but only included mutations in three genes. Building on the ELN’s approach, Patel *et al*. developed an integrated prognostic model with a wider range of molecular markers, using data from patients enrolled in a multicenter randomized controlled trial. Despite the use of this model in clinical practice by some, it has not been validated in a second cohort of patients.

We studied Patel *et al*.’s IGP model in a cohort of patients from two different institutions who were not treated uniformly, reflecting variations seen in clinical practice. The proportions of ECOG 1900 patients in each IGP risk group were similar to those of our study cohort [14]. Yet, the IGP model was not completely validated in our cohort: it did not consistently define groups of AML patients with statistically significant differences in survival. We would therefore not recommend its use as originally described in routine clinical practice. When we applied the IGP model to our patient cohort, it did reclassify 50 of 137 patients with intermediate cytogenetics as having favorable or unfavorable mutational profiles, thus reducing the number of patients with intermediate-risk AML from 70% to 44%. However, not all patients who were reclassified to the favorable and unfavorable risk groups had survival outcomes that were truly different from the intermediate risk group. It is crucial that the risk groups defined by a classification model accurately describe survival outcomes and fully consider all treatment options, including allogeneic stem cell transplant, which we are not able to assess due to lack of available data. In our study, we created refined subgroups, defined by the M-IGP model, to provide a comparison to the previous work by Patel et al. Further studies should investigate appropriate management of these subgroups. It is also possible that a de novo approach to prognostication would in fact define only two prognostic groups (favorable and unfavorable). Further studies using an unbiased approach will address this question.

As shown, we assessed the survival curves for each IGP mutational subgroup to determine where the IGP model was successful and whether it could be refined. We found that patients who had *FLT3*-ITD negative *NPM1*mut/*IDH*mut AML had an OS curve that was significantly different from that of the intermediate IGP risk group and similar to that of patients with favorable cytogenetics, indicating that this molecular subgroup is truly a low-risk group. However, while survival among these patients was similar to survival among patients with favorable cytogenetics in the first two years after diagnosis, it was closer to survival for the intermediate IGP risk group after two years. This may suggest that while patients with *NPM1*mut/*IDH*mut AML respond favorably to induction chemotherapy, they are more likely to relapse than patients with core binding factor AML, leading to long-term outcomes similar to patients with intermediate-risk AML. It is still not clear whether these patients should receive the same management as patients with favorable cytogenetics [26]. Future research should study long-term survival among patients with this mutational profile.

Patients who had *FLT3*-ITD negative AML with co-occurring high-risk mutations (*TET2*, *ASXL1* and/or *PHF6*) had survival similar to patients with intermediate IGP risk AML and therefore did not merit reclassification into an unfavorable risk group. To try to explain this difference from Patel *et al*.’s results, it would be desirable to compare the clinical characteristics of this group in the ECOG 1900 cohort and our study cohort; however, this information was not available for the ECOG 1900 cohort. In contrast, a third group of patients, those with *FLT3*-ITD positive AML and co-occurring high-risk genetic changes (trisomy 8, *TET2* and/or *DNMT3A*) had an OS curve that was significantly different from that of the intermediate IGP risk group and similar to that of patients with unfavorable cytogenetics. This result suggests that this molecular subgroup is truly a high-risk group with an outcome similar to AML with unfavorable cytogenetics. The genes included in this molecular subgroup should be incorporated into clinical testing outside of clinical trials, as these patients may benefit from SCT in first remission, similar to patients with unfavorable cytogenetics.

Although we have not validated the overall IGP model, we do believe that two of the molecular groups identified by Patel *et al*. deserve to be considered in patient management. We therefore refined the IGP model by reclassifying only two out of three mutational profiles. We found that the intermediate M-IGP risk group included a larger proportion of patients (55%) compared to the intermediate IGP risk group (44%), thus increasing the number of patients for whom prognosis is less certain. However, this classification model ensures that patients seen in clinical practice who meet criteria for each risk group are correctly classified. The M-IGP model is thus more accurate when applied to our study cohort, but should be validated in other cohorts in further studies. We propose that the IGP model should not be used indiscriminately in all patient populations, as the performance of prognostic models may vary among patients not included in the cohorts used to establish the models.

Several differences between our study and that conducted by Patel *et al*. [14] may explain why the IGP model was not completely reproducible in our study cohort. First, the methodology used to assess for mutations differed among the Penn, TCGA and ECOG 1900 cohorts. The TCGA and Penn mutational analyses were performed using next generation sequencing methodologies, which are quite analytically sensitive. The current level for clinical reporting at Penn is 4% whereas Sanger sequencing, used by Patel *et al*., is considered to have a sensitivity of only 20%. Second, our group of patients was not treated uniformly with high-dose anthracycline-containing regimens, reflecting differences in clinical practice within and across institutions. Variations in treatment may particularly impact patients with *FLT3*-ITD negative, *NPM1*mut/*IDH*mut AML. Half of the patients in ECOG 1900 were randomized to high-dose daunorubicin induction chemotherapy and recent studies suggest that high-dose chemotherapy could significantly improve prognosis among patients with *NPM1* mutations [27]. Third, the cytogenetic classification schema used by Patel *et al*. is different from the one used for our cohort. We chose to use the MRC schema, as it was developed from a large cohort, is newer, and is commonly used in clinical practice, both in the United States and in Europe [2]. Despite these differences, we found that patients in the study cohort who had intermediate cytogenetics and were *FLT3*-ITD negative *NPM1*mut/*IDH*mut had a very good prognosis, as predicted by the IGP model. Patients who had intermediate cytogenetics and were *FLT3*-ITD positive with co-occurring high-risk genetic changes had a very poor prognosis. This suggests that reclassification of these two groups is valid and reproducible, regardless of differences in methodology and clinical characteristics.

Our study has several limitations. First, it is limited by sample size, particularly when assessing the association between individual mutations and OS. Second, we neither assessed the impact of post-remission therapy and SCT on prognosis, nor included complete remission as an endpoint, as this information was not available for TCGA patients and was not included by Patel *et al*. as part of their published analysis. Third, in the years since Patel *et al*.’s paper was published, increasing evidence has shown that mutations in *CEBPA* generally only affect prognosis when they are biallelic [4,5]. We included both monoallelic and biallelic *CEBPA* mutations in our analysis, to emulate Patel *et al*.’s analysis. Finally, our gene panel did not include *MLL*-PTD, a variant previously shown to be associated with poor prognosis [25,28,29]. However, this mutation is only seen in 5–6% of patients with AML so it is unlikely that its inclusion would have changed our overall conclusions [14,28].

In conclusion, we believe that while molecular markers have the potential to improve risk stratification for patients ≤60 years old with de novo AML, we should exercise caution when developing integrated prognostic models to ensure that patients labeled as having favorable-, intermediate-, or unfavorable-risk AML do indeed have different overall survival. Although we did not completely validate the IGP model, we demonstrated that incorporation of six out of nine mutations (*FLT3*-ITD, *DNMT3A*, *TET2*, *NPM1*, *IDH1*, *IDH2*) into clinical testing at diagnosis can be used to identify a group of lower risk and very high-risk patients with intermediate cytogenetics. As these mutations are strong molecular determinants of survival, incorporation of testing for them among younger patients with AML may help guide therapy. Given that 28% of younger patients with AML have a three-year survival probability of 16%, further studies should be undertaken to develop novel approaches to therapy for the highest-risk patients.

## Supporting Information

S1 Fig Overall survival by cytogenetic risk (n = 197). The survival curve for patients with favorable cytogenetics was significantly different from the survival curve for patients with unfavorable cytogenetics (adjusted p = 0.003). There was no significant difference in OS survival curves between patients with favorable cytogenetics and patients with intermediate cytogenetics (adjusted p = 0.141), or between patients with unfavorable cytogenetics and patients with intermediate cytogenetics (adjusted p = 0.976).
(TIF)

S1 Table Genomic regions targeted in the next-generation sequencing panel for 33 hematologic malignancy-associated genes plus *CEBPA* at the University of Pennsylvania. (DOCX)
