# Supplementary figures and images for: A Modified Integrated Genetic Model for Risk Prediction in Younger Patients with Acute Myeloid Leukemia
Source: PLoS One. 2016 Apr 6;11(4):e0153016. doi: 10.1371/journal.pone.0153016 (PMC4822876; doi:10.1371/journal.pone.0153016)

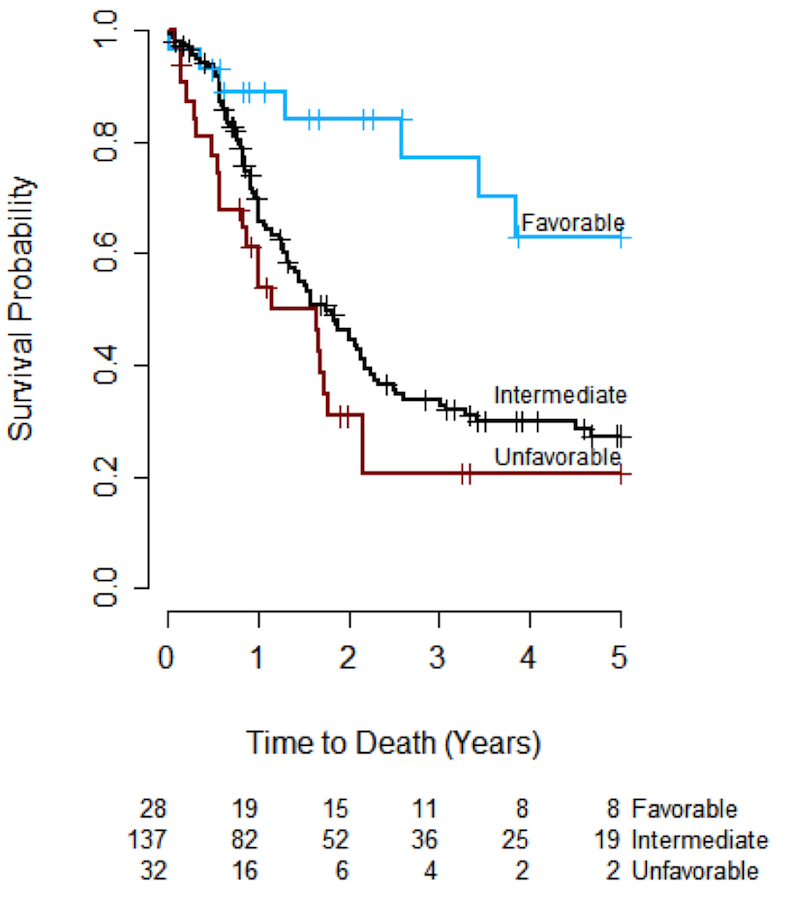

Supplement: S1 Fig — The survival curve for patients with favorable cytogenetics was significantly different from the survival curve for patients with unfavorable cytogenetics (adjusted p = 0.003). There was no significant difference in OS survival curves between patients with favorable cytogenetics and patients with intermediate cytogenetics (adjusted p = 0.141), or between patients with unfavorable cytogenetics and patients with intermediate cytogenetics (adjusted p = 0.976). (TIF) [file pone.0153016.s001.tif]
