# Supplementary material for: A Modified Integrated Genetic Model for Risk Prediction in Younger Patients with Acute Myeloid Leukemia
Source: PLoS One. 2016 Apr 6;11(4):e0153016. doi: 10.1371/journal.pone.0153016 (PMC4822876; doi:10.1371/journal.pone.0153016)
Supplement: S1 Table — (DOCX) [file pone.0153016.s002.docx]

**S1 Table. Genomic regions targeted in the next-generation sequencing panel for 33 hematologic malignancy-associated genes plus *CEBPA* at the University of Pennsylvania**

| **Gene** | **Chromosome** | **Start Position** | **End Position** |
| --- | --- | --- | --- |
| *NPM1* | 5 | 170837513 | 170837705 |
| *DNMT3A* | 2 | 25457144 | 25457322 |
|  | 2 | 25458574 | 25458752 |
|  | 2 | 25459803 | 25460007 |
|  | 2 | 25461997 | 25462221 |
|  | 2 | 25463070 | 25463247 |
|  | 2 | 25463241 | 25463423 |
|  | 2 | 25463422 | 25463643 |
|  | 2 | 25466666 | 25466839 |
|  | 2 | 25466815 | 25466991 |
|  | 2 | 25466989 | 25467209 |
|  | 2 | 25467207 | 25467389 |
|  | 2 | 25468120 | 25468340 |
|  | 2 | 25468969 | 25469153 |
|  | 2 | 25469152 | 25469359 |
|  | 2 | 25469487 | 25469705 |
|  | 2 | 25469908 | 25470124 |
|  | 2 | 25470458 | 25470634 |
|  | 2 | 25470805 | 25471024 |
|  | 2 | 25471023 | 25471216 |
|  | 2 | 25472524 | 25472712 |
|  | 2 | 25497808 | 25498010 |
|  | 2 | 25498366 | 25498537 |
|  | 2 | 25505158 | 25505333 |
|  | 2 | 25505331 | 25505517 |
|  | 2 | 25505516 | 25505689 |
|  | 2 | 25523006 | 25523230 |
|  | 2 | 25536780 | 25536954 |
| *FLT3* | 13 | 28592603 | 28592792 |
|  | 13 | 28608214 | 28608428 |
| *TET2* | 4 | 106154954 | 106155126 |
|  | 4 | 106155101 | 106155270 |
|  | 4 | 106155240 | 106155412 |
|  | 4 | 106155386 | 106155556 |
|  | 4 | 106155531 | 106155704 |
|  | 4 | 106155674 | 106155846 |
|  | 4 | 106155816 | 106155984 |
|  | 4 | 106155954 | 106156126 |
|  | 4 | 106156096 | 106156266 |
|  | 4 | 106156236 | 106156406 |
|  | 4 | 106156377 | 106156548 |
|  | 4 | 106156534 | 106156706 |
|  | 4 | 106156704 | 106156880 |
|  | 4 | 106156879 | 106157050 |
|  | 4 | 106157049 | 106157220 |
|  | 4 | 106157218 | 106157390 |
|  | 4 | 106157388 | 106157564 |
|  | 4 | 106157562 | 106157732 |
|  | 4 | 106157901 | 106158078 |
|  | 4 | 106158077 | 106158260 |
|  | 4 | 106158259 | 106158448 |
|  | 4 | 106158447 | 106158658 |
|  | 4 | 106162452 | 106162636 |
|  | 4 | 106163986 | 106164163 |
|  | 4 | 106164626 | 106164811 |
|  | 4 | 106164810 | 106164999 |
|  | 4 | 106180773 | 106180978 |
|  | 4 | 106182882 | 106183056 |
|  | 4 | 106190766 | 106190963 |
|  | 4 | 106193620 | 106193793 |
|  | 4 | 106193789 | 106193991 |
|  | 4 | 106196104 | 106196285 |
|  | 4 | 106196283 | 106196465 |
|  | 4 | 106196463 | 106196639 |
|  | 4 | 106196638 | 106196815 |
|  | 4 | 106196813 | 106196991 |
|  | 4 | 106196990 | 106197183 |
|  | 4 | 106197181 | 106197377 |
|  | 4 | 106197375 | 106197559 |
|  | 4 | 106197558 | 106197751 |
| *IDH1* | 2 | 209112993 | 209113165 |
|  | 2 | 209113164 | 209113365 |
| *CEBPA* | 19 | 33792150 | 33793350 |
| *RUNX1* | 21 | 36171594 | 36171776 |
|  | 21 | 36206322 | 36206508 |
|  | 21 | 36206507 | 36206688 |
|  | 21 | 36206686 | 36206878 |
|  | 21 | 36206877 | 36207080 |
|  | 21 | 36231530 | 36231711 |
|  | 21 | 36231710 | 36231907 |
|  | 21 | 36252850 | 36253044 |
|  | 21 | 36259040 | 36259218 |
|  | 21 | 36259217 | 36259428 |
|  | 21 | 36265195 | 36265364 |
|  | 21 | 36421136 | 36421343 |
| *IDH2* | 15 | 90631817 | 90632017 |
| *ASXL1* | 20 | 30954182 | 30954375 |
|  | 20 | 30955528 | 30955717 |
|  | 20 | 30956815 | 30957022 |
|  | 20 | 31015830 | 31016003 |
|  | 20 | 31015991 | 31016165 |
|  | 20 | 31016164 | 31016355 |
|  | 20 | 31017140 | 31017343 |
|  | 20 | 31017700 | 31017922 |
|  | 20 | 31019032 | 31019210 |
|  | 20 | 31019208 | 31019398 |
|  | 20 | 31019397 | 31019572 |
|  | 20 | 31020679 | 31020885 |
|  | 20 | 31020986 | 31021161 |
|  | 20 | 31021159 | 31021337 |
|  | 20 | 31021335 | 31021511 |
|  | 20 | 31021510 | 31021729 |
|  | 20 | 31022139 | 31022311 |
|  | 20 | 31022287 | 31022457 |
|  | 20 | 31022432 | 31022607 |
|  | 20 | 31022577 | 31022751 |
|  | 20 | 31022721 | 31022893 |
|  | 20 | 31022871 | 31023037 |
|  | 20 | 31023007 | 31023179 |
|  | 20 | 31023153 | 31023323 |
|  | 20 | 31023293 | 31023467 |
|  | 20 | 31023438 | 31023609 |
|  | 20 | 31023583 | 31023757 |
|  | 20 | 31023727 | 31023905 |
|  | 20 | 31023904 | 31024081 |
|  | 20 | 31024079 | 31024253 |
|  | 20 | 31024251 | 31024425 |
|  | 20 | 31024423 | 31024593 |
|  | 20 | 31024591 | 31024785 |
|  | 20 | 31024783 | 31024957 |
|  | 20 | 31024956 | 31025147 |
| *WT1* | 11 | 32413515 | 32413722 |
|  | 11 | 32417801 | 32418025 |
| *KIT* | 4 | 55593579 | 55593764 |
|  | 4 | 55593888 | 55594067 |
|  | 4 | 55594065 | 55594241 |
|  | 4 | 55594240 | 55594429 |
|  | 4 | 55599230 | 55599445 |
| *TP53* | 17 | 7572916 | 7573135 |
|  | 17 | 7576615 | 7576799 |
|  | 17 | 7576798 | 7576981 |
|  | 17 | 7576980 | 7577167 |
|  | 17 | 7577477 | 7577656 |
|  | 17 | 7578078 | 7578257 |
|  | 17 | 7578255 | 7578433 |
|  | 17 | 7578432 | 7578629 |
| *PHF6* | X | 133511622 | 133511836 |
|  | X | 133511833 | 133512050 |
|  | X | 133512049 | 133512266 |
|  | X | 133527506 | 133527711 |
|  | X | 133527911 | 133528119 |
|  | X | 133547423 | 133547644 |
|  | X | 133547642 | 133547862 |
|  | X | 133547861 | 133548048 |
|  | X | 133548962 | 133549148 |
|  | X | 133549147 | 133549364 |
|  | X | 133551196 | 133551401 |
| *NRAS* | 1 | 115258669 | 115258871 |
| *PTEN* | 10 | 89624217 | 89624439 |
|  | 10 | 89653770 | 89653944 |
|  | 10 | 89685265 | 89685446 |
|  | 10 | 89690783 | 89690991 |
|  | 10 | 89692672 | 89692846 |
|  | 10 | 89692845 | 89693048 |
|  | 10 | 89711791 | 89711969 |
|  | 10 | 89711968 | 89712171 |
|  | 10 | 89717608 | 89717796 |
|  | 10 | 89720552 | 89720731 |
|  | 10 | 89720730 | 89720935 |
|  | 10 | 89725040 | 89725240 |
| *ATM* | 11 | 108098339 | 108098516 |
|  | 11 | 108098515 | 108098718 |
|  | 11 | 108099887 | 108100073 |
|  | 11 | 108106334 | 108106505 |
|  | 11 | 108106504 | 108106685 |
|  | 11 | 108114586 | 108114762 |
|  | 11 | 108114761 | 108114970 |
|  | 11 | 108115415 | 108115601 |
|  | 11 | 108115600 | 108115785 |
|  | 11 | 108117590 | 108117763 |
|  | 11 | 108117762 | 108117947 |
|  | 11 | 108119562 | 108119756 |
|  | 11 | 108119755 | 108119960 |
|  | 11 | 108121327 | 108121494 |
|  | 11 | 108121493 | 108121666 |
|  | 11 | 108121665 | 108121856 |
|  | 11 | 108122468 | 108122642 |
|  | 11 | 108122641 | 108122854 |
|  | 11 | 108123541 | 108123758 |
|  | 11 | 108124449 | 108124639 |
|  | 11 | 108124638 | 108124839 |
|  | 11 | 108126841 | 108127046 |
|  | 11 | 108127045 | 108127260 |
|  | 11 | 108128205 | 108128400 |
|  | 11 | 108129710 | 108129917 |
|  | 11 | 108137894 | 108138112 |
|  | 11 | 108139072 | 108139245 |
|  | 11 | 108139244 | 108139445 |
|  | 11 | 108141766 | 108141965 |
|  | 11 | 108141959 | 108142159 |
|  | 11 | 108143158 | 108143355 |
|  | 11 | 108143329 | 108143505 |
|  | 11 | 108143504 | 108143693 |
|  | 11 | 108150207 | 108150426 |
|  | 11 | 108151622 | 108151812 |
|  | 11 | 108151811 | 108152020 |
|  | 11 | 108153337 | 108153505 |
|  | 11 | 108153504 | 108153689 |
|  | 11 | 108154862 | 108155030 |
|  | 11 | 108155029 | 108155226 |
|  | 11 | 108158326 | 108158503 |
|  | 11 | 108159606 | 108159798 |
|  | 11 | 108159797 | 108160008 |
|  | 11 | 108160290 | 108160469 |
|  | 11 | 108160468 | 108160673 |
|  | 11 | 108163257 | 108163444 |
|  | 11 | 108163443 | 108163664 |
|  | 11 | 108163986 | 108164164 |
|  | 11 | 108165647 | 108165864 |
|  | 11 | 108167989 | 108168182 |
|  | 11 | 108170432 | 108170651 |
|  | 11 | 108172366 | 108172559 |
|  | 11 | 108173479 | 108173654 |
|  | 11 | 108173653 | 108173852 |
|  | 11 | 108175326 | 108175502 |
|  | 11 | 108175501 | 108175694 |
|  | 11 | 108178619 | 108178790 |
|  | 11 | 108180787 | 108180959 |
|  | 11 | 108180958 | 108181125 |
|  | 11 | 108183135 | 108183322 |
|  | 11 | 108186458 | 108186626 |
|  | 11 | 108186606 | 108186790 |
|  | 11 | 108186789 | 108186986 |
|  | 11 | 108188032 | 108188210 |
|  | 11 | 108188209 | 108188412 |
|  | 11 | 108190651 | 108190865 |
|  | 11 | 108192009 | 108192180 |
|  | 11 | 108195936 | 108196115 |
|  | 11 | 108196114 | 108196291 |
|  | 11 | 108196689 | 108196909 |
|  | 11 | 108196908 | 108197125 |
|  | 11 | 108198360 | 108198526 |
|  | 11 | 108200852 | 108201039 |
|  | 11 | 108201038 | 108201241 |
|  | 11 | 108202154 | 108202359 |
|  | 11 | 108202508 | 108202728 |
|  | 11 | 108203470 | 108203649 |
|  | 11 | 108204604 | 108204773 |
|  | 11 | 108205681 | 108205852 |
|  | 11 | 108206523 | 108206728 |
|  | 11 | 108213929 | 108214141 |
|  | 11 | 108216369 | 108216546 |
|  | 11 | 108216545 | 108216746 |
|  | 11 | 108217995 | 108218172 |
|  | 11 | 108224484 | 108224667 |
|  | 11 | 108225535 | 108225738 |
|  | 11 | 108235708 | 108235879 |
|  | 11 | 108235866 | 108236035 |
|  | 11 | 108236034 | 108236239 |
| *CBL* | 11 | 119148777 | 119148980 |
|  | 11 | 119148979 | 119149200 |
|  | 11 | 119149199 | 119149374 |
|  | 11 | 119149373 | 119149556 |
| *ETV6* | 12 | 11803060 | 11803252 |
|  | 12 | 11905381 | 11905600 |
|  | 12 | 11992072 | 11992272 |
|  | 12 | 12006358 | 12006561 |
|  | 12 | 12022258 | 12022432 |
|  | 12 | 12022430 | 12022608 |
|  | 12 | 12022606 | 12022790 |
|  | 12 | 12022789 | 12022996 |
|  | 12 | 12037378 | 12037581 |
|  | 12 | 12038857 | 12039036 |
|  | 12 | 12043860 | 12044073 |
| *KRAS* | 12 | 25380166 | 25380360 |
|  | 12 | 25398201 | 25398388 |
| *PTPN11* | 12 | 112888121 | 112888326 |
|  | 12 | 112892336 | 112892550 |
|  | 12 | 112893753 | 112893946 |
| *XPO1* | 2 | 61719370 | 61719566 |
|  | 2 | 61719565 | 61719766 |
| *SF3B1* | 2 | 198264678 | 198264845 |
|  | 2 | 198264835 | 198265019 |
|  | 2 | 198265018 | 198265225 |
|  | 2 | 198265338 | 198265539 |
|  | 2 | 198265538 | 198265713 |
|  | 2 | 198266023 | 198266206 |
|  | 2 | 198266205 | 198266396 |
|  | 2 | 198266370 | 198266536 |
|  | 2 | 198266534 | 198266710 |
|  | 2 | 198266709 | 198266894 |
|  | 2 | 198267182 | 198267360 |
|  | 2 | 198267359 | 198267558 |
| *GNAS* | 20 | 57484303 | 57484482 |
|  | 20 | 57484481 | 57484684 |
| *mapk1* | 22 | 22123448 | 22123669 |
|  | 22 | 22127160 | 22127350 |
|  | 22 | 22142521 | 22142722 |
| *MYD88* | 3 | 38182246 | 38182430 |
|  | 3 | 38182618 | 38182805 |
| *KLHL6* | 3 | 183273048 | 183273223 |
|  | 3 | 183273222 | 183273405 |
|  | 3 | 183273404 | 183273607 |
| *FBXW7* | 4 | 153245238 | 153245412 |
|  | 4 | 153245411 | 153245616 |
|  | 4 | 153247084 | 153247260 |
|  | 4 | 153247259 | 153247450 |
|  | 4 | 153249359 | 153249556 |
|  | 4 | 153250823 | 153251004 |
|  | 4 | 153251881 | 153252102 |
|  | 4 | 153253745 | 153253938 |
|  | 4 | 153258949 | 153259164 |
|  | 4 | 153268077 | 153268268 |
| *BRAF* | 7 | 140453071 | 140453241 |
|  | 7 | 140481357 | 140481560 |
| *EZH2* | 7 | 148504716 | 148504930 |
|  | 7 | 148506062 | 148506235 |
|  | 7 | 148506233 | 148506409 |
|  | 7 | 148506408 | 148506611 |
|  | 7 | 148507423 | 148507625 |
|  | 7 | 148508713 | 148508919 |
|  | 7 | 148511048 | 148511257 |
|  | 7 | 148512001 | 148512200 |
|  | 7 | 148512594 | 148512780 |
|  | 7 | 148513775 | 148513976 |
|  | 7 | 148523494 | 148523670 |
|  | 7 | 148523669 | 148523870 |
|  | 7 | 148524251 | 148524426 |
|  | 7 | 148525827 | 148526048 |
|  | 7 | 148526817 | 148527024 |
|  | 7 | 148529671 | 148529878 |
|  | 7 | 148543546 | 148543760 |
|  | 7 | 148544225 | 148544430 |
| *JAK2* | 9 | 5073686 | 5073859 |
| *CDKN2A* | 9 | 21968220 | 21968400 |
|  | 9 | 21970801 | 21970989 |
|  | 9 | 21970985 | 21971193 |
|  | 9 | 21971192 | 21971399 |
|  | 9 | 21974375 | 21974548 |
|  | 9 | 21974542 | 21974736 |
|  | 9 | 21974735 | 21974944 |
| *NOTCH1* | 9 | 139390423 | 139390597 |
|  | 9 | 139390567 | 139390749 |
|  | 9 | 139390733 | 139390941 |
|  | 9 | 139390935 | 139391121 |
|  | 9 | 139391119 | 139391303 |
|  | 9 | 139391302 | 139391485 |
|  | 9 | 139391483 | 139391665 |
|  | 9 | 139391664 | 139391839 |
|  | 9 | 139397632 | 139397823 |
|  | 9 | 139399203 | 139399379 |
| *DDX3X* | X | 41196660 | 41196869 |
|  | X | 41204557 | 41204743 |
|  | X | 41204742 | 41204947 |
| *ZMYM3* | X | 70460670 | 70460851 |
|  | X | 70460849 | 70461027 |
|  | X | 70461026 | 70461233 |
|  | X | 70461921 | 70462120 |
|  | X | 70462119 | 70462326 |
|  | X | 70469874 | 70470068 |
|  | X | 70472340 | 70472521 |
|  | X | 70472520 | 70472731 |
